# Using an Adenosine Triphosphate Bioluminescent Assay to Determine Effective Antibiotic Combinations against Carbapenem-Resistant Gram Negative Bacteria within 24 Hours

**DOI:** 10.1371/journal.pone.0140446

**Published:** 2015-10-13

**Authors:** Yiying Cai, Hui Leck, Tze Peng Lim, Jocelyn Teo, Winnie Lee, Li Yang Hsu, Tse Hsien Koh, Thuan Tong Tan, Thean-Yen Tan, Andrea Lay-Hoon Kwa

**Affiliations:** 1 Department of Pharmacy, Singapore General Hospital, Singapore, Singapore; 2 Department of Infectious Diseases, National University Health Systems, Singapore, Singapore; 3 Department of Pathology, Singapore General Hospital, Singapore, Singapore; 4 Department of Infectious Diseases, Singapore General Hospital, Singapore, Singapore; 5 Department of Laboratory Medicine, Changi General Hospital, Singapore, Singapore; 6 Emerging Infectious Diseases, Duke-NUS Graduate Medical School, Singapore, Singapore; 7 Pharmacy, Faculty of Science, National University of Singapore, Singapore, Singapore; Second University of Naples, ITALY

## Abstract

**Background:**

Current *in vitro* combination testing methods involve enumeration by bacterial plating, which is labor-intensive and time-consuming. Measurement of bioluminescence, released when bacterial adenosine triphosphate binds to firefly luciferin-luciferase, has been proposed as a surrogate for bacterial counts. We developed an ATP bioluminescent combination testing assay with a rapid turnaround time of 24h to determine effective antibiotic combinations.

**Methods:**

100 strains of carbapenem-resistant (CR) GNB [30 *Acinetobacter baumannii* (AB), 30 *Pseudomonas aeruginosa* (PA) and 40 *Klebsiella pneumoniae* (KP)] were used. Bacterial suspensions (10^5^ CFU/ml) were added to 96-well plates containing clinically achievable concentrations of multiple single and two-antibiotic combinations. At 24h, the luminescence intensity of each well was measured. Receiver operator characteristic curves were plotted to determine optimal luminescence threshold (T_RLU_) to discriminate between inhibitory/non-inhibitory combinations when compared to viable plating. The unweighted accuracy (UA) [(sensitivity + specificity)/2] of T_RLU_ values was determined. External validation was further done using 50 additional CR-GNB.

**Results:**

Predictive accuracies of T_RLU_ were high for when all antibiotic combinations and species were collectively analyzed (T_RLU_ = 0.81, UA = 89%). When individual thresholds for each species were determined, UA remained high. Predictive accuracy was highest for KP (T_RLU_ = 0.81, UA = 91%), and lowest for AB (T_RLU_ = 0.83, UA = 87%). Upon external validation, high overall accuracy (91%) was observed. The assay distinguished inhibitory/non-inhibitory combinations with UA of 80%, 94% and 93% for AB, PA and KP respectively.

**Conclusion:**

We developed an assay that is robust at identifying useful combinations with a rapid turn-around time of 24h, and may be employed to guide the timely selection of effective antibiotic combinations.

## Introduction

In the past decade, the prescription of effective antimicrobial therapy has been challenged by the rising prevalence of extensively-drug resistant (XDR) and pan-drug resistant (PDR) Gram negative bacteria (GNB) [1]. In addition to drug-resistant non-fermenters such as *Pseudomonas aeruginosa* and *Acinetobacter baumannii*, the rapid emergence of carbapenem-resistant (CR) Enterobacteriaceae represents an added threat to the existing antibiotic armamentarium [1,2]. As last-line broad-spectrum antibiotics such as carbapenems are rendered useless, combination antibiotic therapy has been increasingly accepted as common practice in the treatment of XDR- and PDR-GNB infections [3].

Knowledge of the *in vitro* susceptibility of a pathogen has been the mainstay for guiding clinicians in the selection of antibiotics [4]. Unfortunately, traditional single-antibiotic susceptibility testing methods have limited utility when predicting the efficacy of antibiotic combinations against XDR- or PDR-GNB[4]. While other *in vitro* combination testing methods such as the time-kill studies have been employed to predict effective combinations, these methods require enumeration using viable plate count and are cumbersome, time-consuming and labor-intensive, and are unlikely to provide results in a timely manner for routine clinical use. Hence, a rapid susceptibility testing method that can identify effective antibiotic combinations with a sufficiently rapid turnaround time is urgently needed.

The use of bacterial adenosine triphosphate (ATP) as a surrogate measure for bacterial load has been previously suggested as an alternative to enumeration via viable plating [5–7]. ATP is the principal energy carrier of all living organisms. It is ubiquitously present in all living bacterial cells, and is rapidly lost from dead cells [8]. Measurement of ATP levels can be indirectly achieved using the luciferase-luciferin reaction. When the enzyme luciferase, extracted from fireflies of the genus *Photinus*, and its substrate luciferin is added to an ATP-containing sample, ATP is converted to adenosine monophosphate (AMP) with emission of bioluminescence in the wavelength range of 470–700nm (peak wavelength 562nm) [8]. The amount of bioluminescence emitted can be quantified using a luminometer, and the amount of light emitted during the reaction has been shown to be directly proportional to the amount of ATP present in the sample [6]. The reaction is described in the equation shown below:

$$\begin{array}{l} D\;luciferin+ATP+O_{2}\overset{Luciferin}{\underset{Mg^{2+}}{\to }}Oxyluciferin+AMP+pp_{i}+h\nu \end{array}$$

To date, a small number of studies have employed the use of ATP bioluminescence in antimicrobial susceptibility testing against single antimicrobial agents [6,7,9,10]. Hattori *et al* utilized ATP bioluminescence to determine the susceptibility of Gram negative and Gram positive bacteria against different antimicrobial agents [7,10]. In another study by Kapoor *et al*, ATP bioluminescence was employed to test the susceptibility of rapidly growing mycobacteria against biocides [7]. Result from both studies demonstrated high overall accuracy compared to conventional susceptibility testing methods, suggesting that ATP bioluminescence-based methods could potentially be used in place of conventional susceptibility testing against single antibiotics or biocides [6,7,10].

Based on findings from previous studies, we hypothesized that ATP bioluminescence may be useful in determining effective antibiotic combinations against CR-GNB in a rapid manner suitable for routine clinical use. Hence, the objective of our current study is to firstly, develop a multiple antibiotic combination testing assay using ATP bioluminescence to identify effective antibiotic combinations against CR-GNB with a rapid turn-around time of 24h, and secondly, prospectively validate the predictive accuracy of the assay compared to viable plating methods using additional CR-GNB strains.

## Materials and Methods

### Microorganisms and Susceptibility Testing

Clinical strains of carbapenem-resistant (CR) *A*. *baumannii* (n = 30), *P*. *aeruginosa* (n = 30) and *K*. *pneumoniae* (n = 40) were collected from Singapore hospitals from 2009–13 to develop the ATP bioluminescence assay. Genus identity was determined using Vitek 2 ID-GN cards (bioMerieux, Inc., Hazelwood, MO). Carbapenem susceptibility was determined using disk diffusion and interpreted in accordance to the Clinical and Laboratory Standards Institute (CLSI) guidelines [11]. MICs to multiple antibiotics were performed using custom-made microbroth dilution panels (Trek Diagnostics, East Grinstead, UK), and susceptibility defined based on CLSI breakpoints [11]. All isolates were stored at -80°C in CryoCare bacteria preservers (Key Scientific Products, Round Rock, TX), and fresh isolates were sub-cultured twice on 5% blood agar plates (Biomedia-Bloxwich, Malaysia) for 24 h at 35°C prior to each experiment.

### Resistance Mechanisms

All *A*. *baumannii* isolates were screened for *bla*_OXA-23_, *bla*_OXA-24_, *bla*_OXA-51_, and *bla*_OXA-58_ genes using a multiplex PCR assay [12]. For *P*. *aeruginosa* and *K*. *pneumoniae* isolates, a multiplex PCR assay with five different primer pairs was employed to detect genes encoding commonly acquired metallo-β-lactamases (MBLs) (*bla*_VIM_, *bla*_IMP_, *bla*_SIM_, *bla*_GIM_, *bla*_SPM_) [13]. In addition, determination of genes encoding ESBLs, plasmid-mediated AmpCs, NDM and KPCs were performed using PCR for *K*. *pneumoniae* [13,14]. Changes in porin gene expression (OmpK35 and OmpK36) were determined for *K*. *pneumoniae* using reverse transcriptase (RT) PCR, and presence of efflux pumps was determined using efflux pump inhibitor phenyl-arginine-β-naphthylamide (PAβN) (50μg/ml) [15,16].

### Antimicrobial Agents

A total of six antibiotics were employed for antibiotic combination testing, at concentrations shown in Table 1 [17–22]. Amikacin, polymyxin B and rifampicin were obtained from Sigma-Aldrich (St. Louis, MO). Meropenem was provided by Astra Zeneca Inc. Tigecycline was provided by Wyeth Pharmaceuticals. Levofloxacin was provided by Daiichi Sankyo Co. Stock solutions of all antimicrobial agents except rifampicin were prepared in sterile water. Rifampicin was dissolved in dimethyl sulfoxide (DMSO) and was then serially diluted in sterile water to the desired concentration. The final DMSO concentration (<1% v/v) had no effect on bacterial growth [11,23].

**Table 1:** Simulated antibiotic dosing regimens and corresponding drug concentrations.

Antibiotics^*a*^	Simulated Dosing Regimens	Concentration (mg/L)
Amikacin	15–20 mg/kg every 24 hours	65
Levofloxacin	750mg every 24 hours	8
Rifampicin	IV/PO 600mg every 12 hours	2
Polymyxin B	25,000 to 30,000IU/kg/day	2
Tigecycline^*b*^	100mg every 12 hours	2
Meropenem	2g every 8 hours (infused over 3 hours)	20

^*a*^ Concentrations shown represented clinically achievable unbound serum concentrations for all listed antibiotics at the corresponding doses stated except tigecycline.

^*b*^ Concentration shown represented average tissue concentration at the corresponding dose stated.

Against *A*. *baumannii*, polymyxin B, tigecycline and rifampicin alone and in two-drug combinations were tested. Against *P*. *aeruginosa* and *K*. *pneumoniae*, polymyxin B, amikacin, meropenem alone and in two-drug combinations; and polymyxin B, meropenem, tigecycline, rifampicin and levofloxacin alone and in two-drug combinations were respectively tested. These combinations were selected based on their promising activity against the respective organisms in previous studies [12,24–26].

### Measurement of bacterial ATP

ATP was quantified using the BacTiter-Glo microbial viability assay (Promega, Madison, WI). To quantify bacterial ATP, the microbial viability assay reagent was first prepared according to the manufacturer’s instructions. 100μl BacTiter-Glo assay reagent was added to 100μl of the test sample (of which ATP content is to be measured), and incubated for 15 min at room temperature, to release bacterial ATP and generate a bioluminescent signal. Bioluminescent light, measured as relative light units (RLU), was recorded by a GloMax Integrated Luminescence System (Promega, Madison, WI), with a 1-s integration time. Background RLU values, determined using sterile MHB, were then subtracted from the RLU values obtained at 0h and at 24h.

### Relationship between Viable Counts and ATP Bioluminescence

To demonstrate the relationship between viable counts and ATP bioluminescence in each of the three Gram negative organisms, calibration curves (log_10_-corrected RLU/100μl against log_10_ CFU/ml) were plotted. Three clinical CR *A*. *baumannii*, *P*. *aeruginosa* and *K*. *pneumoniae* strains (AB 112, PA 14004 and KP 53879) and three American Type Culture Collection (ATCC) strains (*A*. *baumannii* ATCC 19606, *P*. *aeruginosa* ATCC 27853 and *K*. *pneumoniae* ATCC 13883) were employed for calibration. Approximately 10^7^ CFU/ml of each strain was suspended in cation-adjusted Mueller Hinton Broth (Ca-MHB) (BBL, BD, USA). The inoculum was further diluted serially 10-, 100-, 1,000- and 10,000-fold to achieve concentrations of approximately 10^3^ to 10^7^ CFU/ml. Total bacterial count was quantified by depositing serial ten-fold dilutions of the broth sample onto Mueller-Hinton Agar (MHA) plates, incubated at 35°C for 18 to 24h, and enumerated visually. In addition, the ATP content in each sample was determined using the bioluminescent assay as described above.

### Bioluminescent Antibiotic Combination Testing

Bioluminescent combination antibiotic testing was performed in 96-well flat-bottom white microtiter plates (Greiner Bio-One, Frickenhausen, Germany) containing 50μl of test antibiotic(s) per well. Overnight bacterial cultures were diluted with pre-warmed Ca-MHB and incubated at 35°C until log-phase growth. The bacterial suspension was then diluted with Ca-MHB according to absorbance 630nm and 50μl added to each well, This gave a final bacterial concentration of approximately 10^5^ CFU/ml (1 × 10^5^ CFU/ml to 5 × 10^5^ CFU/ml) in each well. Growth and sterility wells were included. The wells were covered with a sealing film and incubated with agitation at 35°C for 24h. At 24h, the total ATP content in each well was determined using bioluminescent assay. The assay was repeated on the same and different days, to ensure intra-day and inter-day reproducibility of results.

### Combination Testing via Enumeration of Viable Counts

Combination testing was carried out in 96-well round bottom clear microtiter plates (Greiner Bio-One, Frickenhausen, Germany) containing 100μl of test antibiotic(s) per well, modified from a method published by Aaron *et al* [27]. Overnight bacterial cultures were diluted with pre-warmed Ca-MHB and incubated at 35°C until log-phase growth. The bacterial suspension was prepared as stated above, and 100μl added to each well to achieve final bacterial concentration of approximately 10^5^ CFU/ml (1 × 10^5^ CFU/ml to 5 × 10^5^ CFU/ml). Growth and sterility wells were included. The wells were covered and incubated with agitation at 35°C for 24h.

At 24h, the contents of each well were sampled and measured to ensure <10% loss in volume. The measured contents were centrifuged at 10,000 × g for 15 minutes and the pellet reconstituted with sterile normal saline to original volume to minimize drug carry-over. Total bacterial count was quantified by depositing serial ten-fold dilutions of the sample onto MHA plates as described above. The lower limit of detection for the colony counts were 400 CFU/ml.

### Establishing Thresholds to Discriminate Inhibitory/Non-inhibitory Combinations (Internal Validation)

All data was analyzed using the SPSS Version 17.0.1 software (SPSS Inc., Chicago IL, USA). Firstly, background RLU values were subtracted from the RLU values obtained at 0h and at 24h. The log_10_-corrected RLU at time zero was then subtracted from the log_10_-corrected RLU obtained at 24h, to estimate change in log_10_-corrected RLU values from initial inoculum (*Δ*RLU). A receiver operating characteristic (ROC) curve analysis was carried out for each species, using these log_10_-scale differences, to establish the most optimal thresholds (T_RLU_) to discriminate between inhibitory and non-inhibitory combinations as determined by viable plating. This meant that samples with ΔRLU less than or equals to the corresponding T_RLU_ value would be classified as “inhibitory, and samples with ΔRLU more than its corresponding T_RLU_ value would be classified as “non-inhibitory”. The T_RLU_ values were chosen to maximize the unweighted classification accuracy–this was akin to finding the tangent point to a 45° line on ROC curve, hence assigning an equal importance to sensitivity and specificity. Separate analyses were carried out for each species (all antibiotics and individual antibiotic combinations), as the relationship may differ across species and antibiotics.

### External Validation of ATP bioluminescence assay

To further validate the established T_RLU_ thresholds for each organism and antibiotic combination, additional strains of CR *A*. *baumannii* (n = 15), strains of CR *P*. *aeruginosa* (n = 15) and strains of CR *K*. *pneumoniae* (n = 20) were prospectively collected from Singapore hospitals from 2013–2014. Combination testing via determination of viable counts and ATP bioluminescent assay method were carried out as described above. Using the ATP bioluminescent results and the previously established T_RLU_ values, each sample was then classified as “inhibitory” or “non-inhibitory”. These classification results were compared to results obtained from viable counts method for agreement, with viable counts method as the reference method. The sensitivity, specificity and the unweighted classification accuracy of the ATP bioluminescent assay method was determined for each species and antibiotic combination.

### Definitions

Extensively-drug resistance was defined as non-susceptibility to at least one agent in all but two or fewer antimicrobial categories [28]. Pan-drug resistance was defined as non-susceptibility to all agents in all antimicrobial categories [28]. Evidence of an at least inhibitory activity was defined as any decrease in colony count on subculture of an organism in the presence of antibiotics compared to initial inoculum at 24h. Sensitivity was defined by the formulae *TP / (TP + FN)*, where TP was the number of samples with inhibitory activity as determined by viable plating with ΔRLU ≤ T_RLU_, and FN was the number of samples with inhibitory activity as determined by viable plating and ΔRLU > T_RLU_. Specificity was defined by the formulae *TN / (TN + FP)*, where TN was the number of samples with non-inhibitory activity as determined by viable plating with ΔRLU > T_RLU_, and FP was number of samples with non-inhibitory activity as determined by viable plating and ΔRLU ≤ T_RLU._ Unweighted accuracy was defined as (*TP + TN) /N*, where N is the total number of samples included into each analysis.

### Ethics Statement

This study was approved by the SingHealth institutional ethics review board prior to initiation (2012/110/D).

## Results

### Susceptibility Testing and Resistance Mechanisms

Of the 100 CR-GNB isolates employed to develop the ATP bioluminescence assay, 82 were XDR and 18 were PDR (two *A*. *baumannii*, nine *P*. *aeruginosa* and seven *K*. *pneumoniae*). All isolates were not susceptible to meropenem, imipenem, ciprofloxacin, piperacillin/tazobactam, ceftazidime and cefepime *(data not shown)* [11]. MIC_50_ and MIC_90_ for polymyxin B for *A*. *baumannii*, *P*. *aeruginosa* and *K*. *pneumoniae* were 1mg/l and 2mg/l, 2mg/l and 4mg/l, and 2mg/l and 4mg/l respectively. There are no current CLSI susceptibility break points for tigecycline against *A*. *baumannii* or *K*. *pneumoniae*.[11] MIC_50_ and MIC_90_ of tigecycline for the *A*. *baumannii* and *K*. *pneumoniae* strains were 4mg/l and 8mg/l, and 2mg/l and 4mg/l respectively.

All *A*. *baumannii* isolates harbored *bla*_OXA-23_ and *bla*_OXA-51_ carbapenemase genes. For *P*. *aeruginosa*, the majority harbored either genes encoding VIM (10/30, 33.3%) or IMP (9/30, 30.0%) metallo-beta-lactamases; in addition, one *P*. *aeruginosa* strain (1/30, 3.3%) harbored *bla*_VEB_. A wide variety of mechanisms mediating carbapenem resistance was observed in *K*. *pneumoniae*. Carbapenemases were responsible for mediating resistance in 35 (87.5%) *K*. *pneumoniae* isolates–*bla*_OXA-181_ genes were most commonly detected (17/40, 42.5%), followed by *bla*_KPC_ (16/40, 40.0%) *bla*_NDM_ (12/40, 30.0%) and *bla*_OXA-48_ genes (7/40, 17.5%). Approximately a quarter (11/40, 27.5%) of the *K*. *pneumoniae* isolates harbored more than one gene encoding carbapenemases; in 10 (25.0%) isolates, both *bla*_NDM_ and *bla*_XOA-181_ resistance genes was detected, while one (2.5%) co-harbored *bla*_NDM_ and *bla*_OXA-48_. In five (12.5%) *K*. *pneumoniae* isolates, ESBLs with reduced expression of porin genes were detected. Addition of PAβN did not result in a decrease in carbapenem MICs in any *K*. *pneumoniae* isolates, suggesting absence of carbapenem efflux.

### Relationship between Viable Counts and ATP Bioluminescence

**Fig 1:**
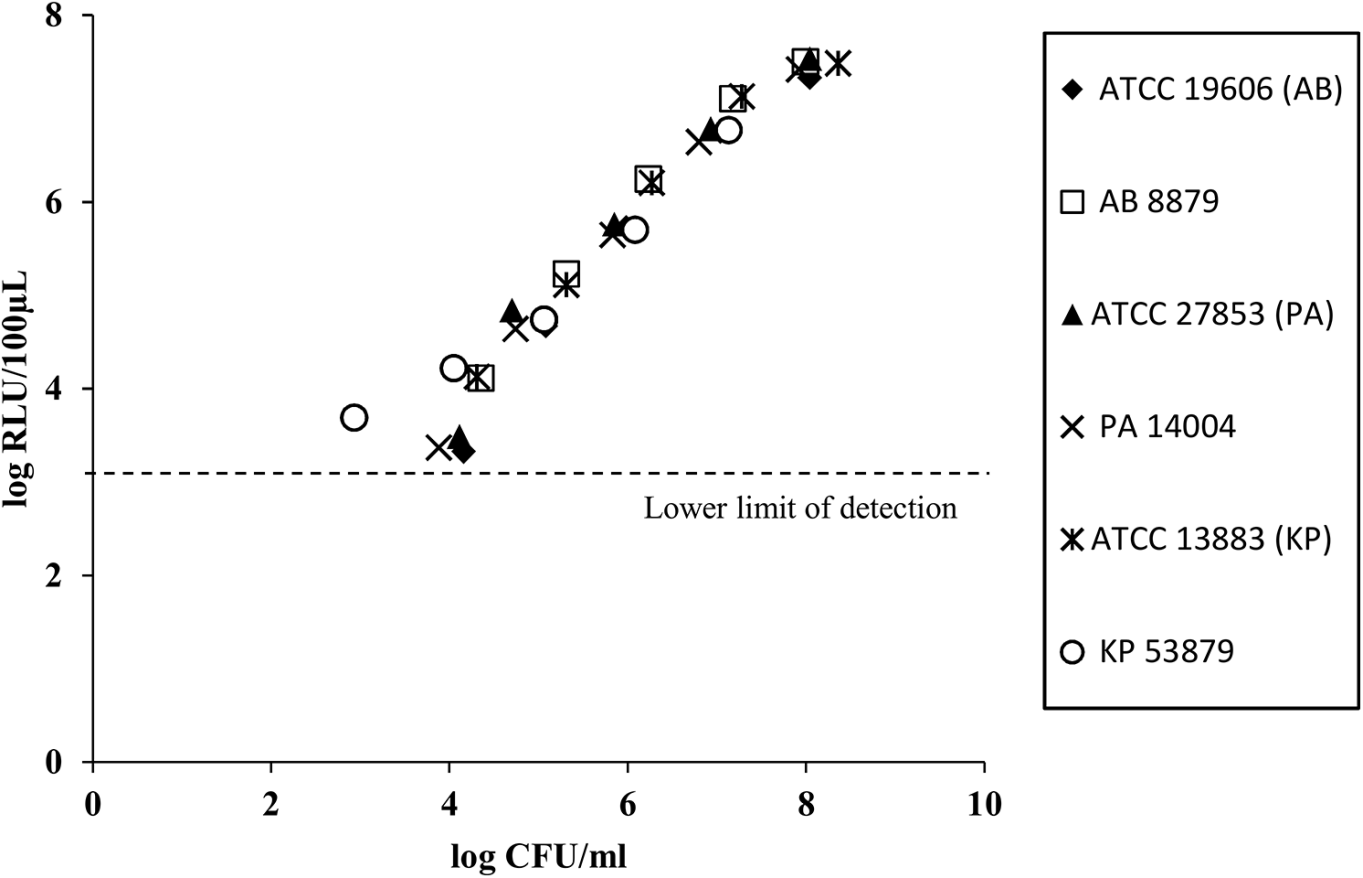
Relationship between bioluminescence (in RLU) and number of organisms determined by viable plate count method. A linear log_10_ RLU/100μl to log_10_ CFU/ml relationship was observed for all six strains, including the carbapenem-resistant GNB strains (r^2^ range: 0.96–0.99). Abbreviations used: AB = *A*. *baumannii*, ATCC = American Type Culture Collection, CFU = colony forming units, GNB = Gram negative bacteria, KP = *K*. *pneumoniae*, PA = *P*. *aeruginosa*, RLU = relative light units.

For all three ATCC strains, the ATP bioluminescence assay displayed a linear relationship between log_10_-corrected RLU values and cell number (CFU/ml) in the operating range of approximately 10^3^ to 10^7^ CFU/ml (r^2^ = 0.96 to 0.98) when the bacteria are suspended in Ca-MHB (Fig 1). This linear relationship was also reproduced in the CR-GNB strains (r^2^ = 0.97 to 0.99) within the same operating range. The lower limit of detection was approximately between 10^3^ RLU/100μl, which corresponded to approximately 10^3^ to 10^4^CFU/ml for all strains; below this, bacterial RLU readings were confounded by the background RLU readings in sterile MHB. The maximum limit of detection was approximately 10^7^ CFU/ml; above 10^7^ CFU/ml, increase in cell number on viable plating did not result in a further increase in RLU values *(data not shown)*.

### Thresholds (T_RLU_) to Discriminate Inhibitory/Non-inhibitory Combinations

**Fig 2:**
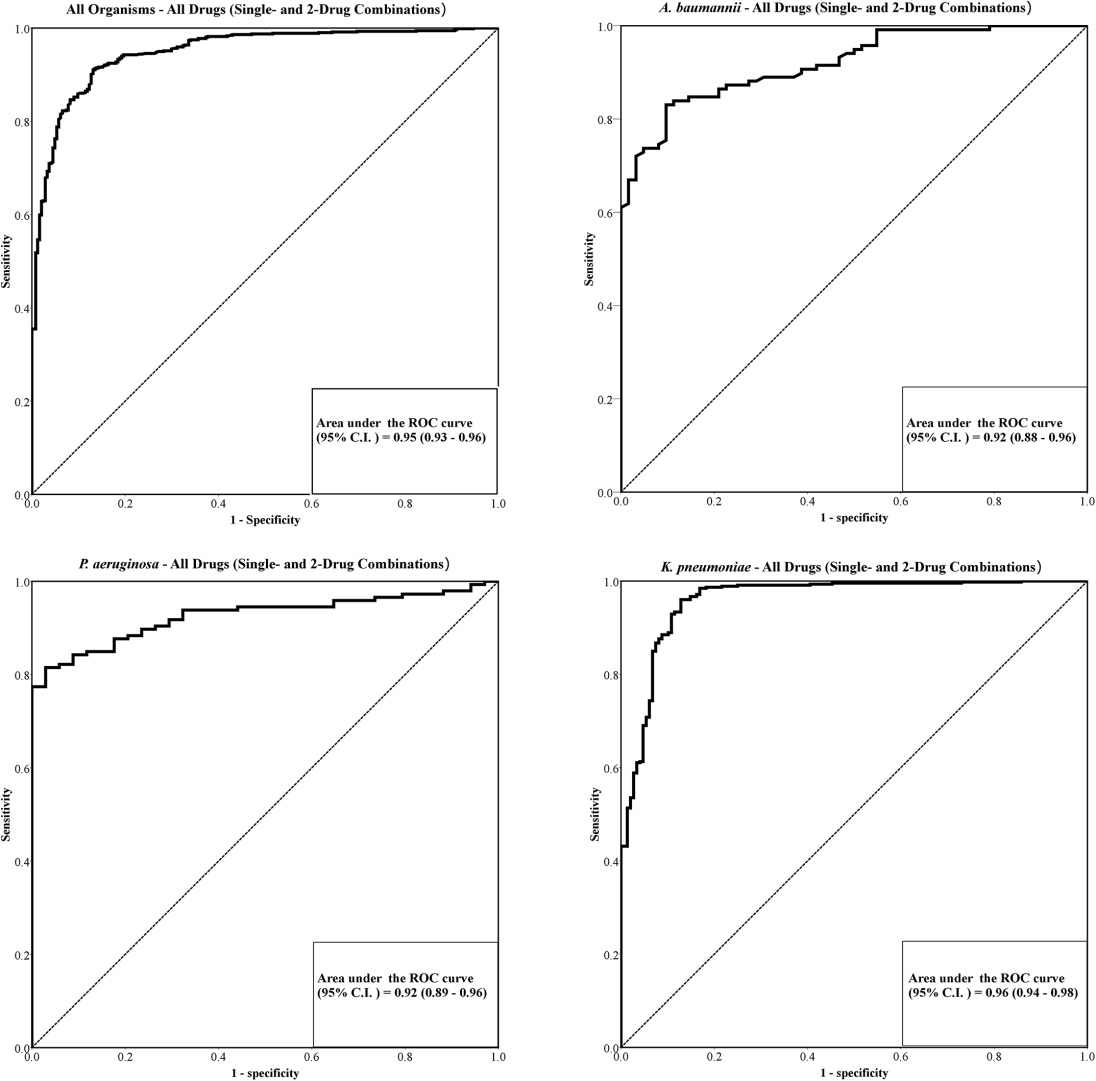
Receiver operator characteristic (ROC) curve for (A) all GNB organisms and antibiotic combinations, (B) *A*. *baumannii*, (C) *P*. *aeruginosa*, and (D) *K*. *pneumoniae*, for single and 2-drug combinations. High area under the ROC curves (0.92–0.96) was observed, signifying high predictive accuracy when bioluminescent assay was compared to the conventional viable plate count method. Abbreviations used: AUC = area under curve, CI = confidence interval, GNB = Gram negative bacteria, ROC = Receiver operator characteristic

The ROC curves for single and 2-drug combinations against all Gram negative organisms, as well as for each organism are shown in Fig 2. To generate the ROC curves to establish the most optimal thresholds to discriminate between inhibitory and non-inhibitory combinations, a total of 960 antibiotic-bacteria observations (180 observations for *A*. *baumannii*, 180 observations for *P*. *aeruginosa* and 600 observations for *K*. *pneumoniae*) were included into the analysis (Fig 2).

Table 2 summarized the results of combination antibiotic testing based on enumeration by viable counting, and accuracy of the ROC curve analysis results by organism and by individual antibiotic combinations. When all GNB organisms and antibiotic combinations were pooled and collectively analyzed, the bioluminescent ATP assay demonstrated high sensitivity, specificity and unweighted classification accuracy for all three species when compared to the conventional plate count method. The overall T_RLU_ value that provided maximum unweighted accuracy was 0.81, which had a sensitivity of 91.1%, specificity of 86.9% and unweighted accuracy of 89.0%. When individualized T_RLU_ values were determined for each species (pooled for all antibiotic combinations), the unweighted accuracy of the established thresholds remained high for all three species (Table 2). Interestingly, the threshold established for *A*. *baumannii* (T_RLU_ = 0.83) and *K*. *pneumoniae* (T_RLU_ = 0.81) were similar to the overall T_RLU_ value, but not for *P*. *aeruginosa* (T_RLU_ = 0.22).

**Table 2:** Establishment of T_RLU_ Thresholds to Discriminate between Inhibitory and Non-Inhibitory Antibiotics for *A*. *baumannii* (n = 30), *P*. *aeruginosa* (n = 30) and *K*. *pneumoniae* (n = 40).

Antibiotics	No. of Specimens Inhibitory/Non-Inhibitory based on Viable Count Determination (%)		Area under the ROC curve (95% CI)^*a*^	Sensitivity and Specificity of Bioluminescence Assay w.r.t Viable Counts (%)^a^		Unweighted Accuracy of the Assay (%)^*a*^	Established T_RLU_ values^a^
	Inhibitory	Non-Inhibitory		Sensitivity	Specificity		
**All Organisms (No. of isolates = 100)**							
All antibiotics (single and 2-drug combinations)	716 (74.6)	244 (25.4)	0.95 (0.93–0.96)	91.1	86.9	89.0	**0.81**
***A*. *baumannii* (No. of isolates = 30)**							
All antibiotics (single and 2-drug combinations)	118 (65.6)	62 (34.4)	0.92 (0.88–0.96)	83.1	90.3	86.7	**0.83**
Polymyxin B	28 (93.3)	2 (6.7)	-	-	-	-	**-**
Rifampicin	3 (10.0)	27 (90.0)	-	-	-	-	**-**
Tigecycline	8 (26.7)	22 (73.3)	-	-	-	-	**-**
Polymyxin B + rifampicin	28 (93.3)	2 (6.7)	0.94 (0.90–0.99)	90.0	93.3	91.7	**0.49**
Polymyxin B + tigecycline	29 (96.7)	1 (3.33)	0.94 (0.88–0.98)	89.2	92.0	90.6	**1.03**
Tigecycline + rifampicin	22 (73.3)	8 (26.7)	0.80 (0.71–0.89)	69.7	71.9	70.8	**1.27**
***P*. *aeruginosa* (No. of isolates = 30)**							
All antibiotics (single and 2-drug combinations)	146 (81.1)	34 (18.8)	0.92 (0.89–0.96)	82.2	97.1	89.7	**0.22**
Polymyxin B	29 (96.7)	1 (3.3)	-	-	-	-	**-**
Amikacin	23 (76.7)	7 (23.3)	-	-	-	-	**-**
Meropenem	7 (23.3)	23 (76.7)	-	-	-	-	**-**
Polymyxin B + amikacin	30 (100)	0 (0)	0.98 (0.96–1.00)	93.9	100	97.0	**0.27**
Polymyxin B + meropenem	30 (100)	0 (0)	0.92 (0.86–0.97)	84.8	87.5	86.2	**0.45**
Amikacin + meropenem	27 (90.0)	3 (10.0)	0.89 (0.81–0.95)	73.7	90.9	82.3	**0.42**
***K*. *pneumoniae* (No. of isolates = 40)**							
All antibiotics (single and 2-drug combinations)	452 (75.3)	148 (24.7)	0.96 (0.94–0.98)	92.9	89.2	91.1	**0.81**
Polymyxin B	37 (92.5)	3 (7.5)	-	-	-	-	**-**
Rifampicin	0 (0)	40 (100)	-	-	-	-	**-**
Tigecycline	20 (50.0)	20 (50.0)	-	-	-	-	**-**
Meropenem	30 (75.0)	10 (25.0)	-	-	-	-	**-**
Levofloxacin	17 (42.5)	23 (57.5)	-	-	-	-	**-**
Polymyxin B + rifampicin	39 (97.5)	1 (2.5)	0.99 (0.99–1.00)	100	97.7	98.9	**1.23**
Polymyxin B + tigecycline	37 (92.5)	3 (7.5)	0.95 (0.91–0.99)	89.4	84.6	87.0	**0.42**
Polymyxin B + meropenem	39 (97.5)	1 (2.5)	0.98 (0.96–1.00)	98.6	92.9	95.8	**1.44**
Polymyxin B + levofloxacin	40 (100)	0 (0)	0.99 (0.98–1.00)	100	94.6	97.3	**1.76**
Rifampicin + tigecycline	35 (87.5)	5 (12.5)	0.92 (0.88–0.97)	92.7	84.6	88.7	**1.20**
Rifampicin + meropenem	32 (80.0)	8 (20.0)	0.99 (0.98–1.00)	96.8	96.6	96.7	**1.26**
Rifampicin + levofloxacin	17 (42.5)	23 (57.5)	1.00 (1.00–1.00)	100	100	100	**1.74**
Tigecycline + meropenem	39 (97.5)	1 (2.5)	0.91 (0.79–0.95)	87.6	80.6	84.1	**0.50**
Tigecycline + levofloxacin	36 (90.0)	4 (10.0)	0.89 (0.82–0.96)	95.9	78.7	87.5	**1.16**
Meropenem + levofloxacin	34 (85.0)	6 (15.0)	0.96 (0.91–1.00)	95.1	92.3	93.7	**1.52**

^*a*^ For each antibiotic pair; results from the single drugs and 2-drug combinations were employed to generate the ROC curve and determine the sensitivity and specificity of the assay.

When separate thresholds were established for each antibiotic combination within each organism, individual T_RLU_ values differed from the overall threshold for each organism, and each antibiotic combination. For instance, the T_RLU_ that discriminated between inhibitory and non-inhibitory combinations for polymyxin B plus rifampicin in *A*. *baumannii* was 0.49; this is in contrast with the T_RLU_ for polymyxin B plus tigecycline (T_RLU_ = 1.03). The accuracy of the bioluminescent ATP assay was also highly dependent on the bacterial species as well as the antibiotic combinations (unweighted accuracy range: 70.8%–100%). Of note, most tigecycline-containing combinations had lower unweighted accuracy than the other combinations–tigecycline plus rifampicin against *A*. *baumannii*, in particular, has the lowest unweighted accuracy (70.8%).

### Prospective Validation of ATP bioluminescence assay

A total of 50 CR-GNB isolates, constituting 480 different antibiotic-bacteria observations (90 observations for *A*. *baumannii*, 90 observations for *P*. *aeruginosa* and 300 observations for *K*. *pneumoniae*) were employed to prospectively validate the bioluminescent assay. Of these, 10 were PDR (2 *A*. *baumannii*, 3 *P*. *aeruginosa* and 5 *K*. *pneumoniae*). MIC_50_ and MIC_90_ of polymyxin B were 2mg/l and ≥16mg/l; MIC_50_ and MIC_90_ of tigecycline for the *A baumannii* and *K*. *pneumoniae* collectively were 4mg/l and 16mg/l respectively. All *A*. *baumannii* strains harbored *bla*_OXA-23_ and *bla*_OXA-51_ genes, while 8 (53.3%) *P*. *aeruginosa* strains harbored genes encoding VIM and IMP metallo-beta-lactamases. *bla*_KPC_ genes were most commonly detected (7/20, 35.0%) in *K*. *pneumoniae*, followed by *bla*_OXA-181_ (4/20, 20.0%) and *bla*_OXA-48_ (4/20, 20.0%).

A high degree of predictive accuracy was observed upon external validation of the previously established thresholds (Table 3). Overall, the ATP bioluminescence assay predicted inhibitory and non-inhibitory combinations with an unweighted accuracy of 91.2% (sensitivity = 90.8%, specificity = 92.9%). Likewise, when individualized T_RLU_ values for each species were validated, the unweighted accuracy of the established thresholds remained high for all three species (unweighted accuracy range: 80.2%–92.6%). Similar to results observed in ROC generation, the accuracy of the assay was also highly dependent on the bacterial species; similarly, tigecycline plus rifampicin yielded the lowest accuracy (unweighted accuracy = 72.7%) upon external validation.

**Table 3:** Prospective Validation of the Established T_RLU_ Thresholds using prospectively collected strains of *A*. *baumannii* (n = 15), *P*. *aeruginosa* (n = 15) and *K*. *pneumoniae* (n = 20).

Antibiotics	No. of Single Drug and 2-drug combinations Inhibitory/Non-Inhibitory based on Viable Count Determination (%)		Sensitivity and Specificity of the Established T_RLU_ Thresholds^*a*^		Unweighted Accuracy of the Established T_RLU_ Thresholds^*b*^
	Inhibitory	Non-Inhibitory	Sensitivity	Specificity	
**All Organisms (No. of isolates = 50)**					
All antibiotics (single and 2-drug combinations)	331 (69.0)	149 (31.0)	90.8	92.9	91.2
***A*. *baumannii* (No. of isolates = 15)**					
All antibiotics (single and 2-drug combinations)	56 (62.2)	34 (37.8)	75.0	85.3	80.2
Polymyxin B	11 (73.3)	4 (26.7)	-	-	-
Rifampicin	3 (20.0)	12 (80.0)	-	-	-
Tigecycline	5 (33.3)	10 (66.7)	-	-	-
Polymyxin B + rifampicin	14 (93.3)	1 (6.7)	89.2	88.2	88.7
Polymyxin B + tigecycline	12 (80.0)	3 (20.0)	85.7	82.3	84.0
Tigecycline + rifampicin	11 (73.3)	4 (26.7)	68.4	76.9	72.7
***P*. *aeruginosa* (No. of isolates = 15)**					
All antibiotics (single and 2-drug combinations)	76 (84.4)	14 (15.6)	86.8	100	93.4
Polymyxin B	14 (93.3)	1 (6.7)	-	-	-
Amikacin	12 (80.0)	3 (20.0)	-	-	-
Meropenem	5 (33.3)	10 (66.7)	-	-	-
Polymyxin B + amikacin	15 (100)	0 (0)	95.1	100	97.6
Polymyxin B + meropenem	15 (100)	0 (0)	85.3	100	92.7
Amikacin + meropenem	15 (100)	0 (0)	78.5	100	89.3
***K*. *pneumoniae* (No. of isolates = 20)**					
All antibiotics (single and 2-drug combinations)	199 (66.3)	101 (33.7)	94.0	91.1	92.6
Polymyxin B	14 (70.0)	6 (30.0)	-	-	-
Rifampicin	0 (0)	20 (100)	-	-	-
Tigecycline	12 (60.0)	8 (40.0)	-	-	-
Meropenem	14 (70.0)	6 (30.0)	-	-	-
Levofloxacin	7 (35.0)	13 (65.0)	-	-	-
Polymyxin B + rifampicin	15 (75.0)	5 (25.0)	96.6	100	98.3
Polymyxin B + tigecycline	17 (85.0)	3 (15.0)	79.1	94.1	86.6
Polymyxin B + meropenem	17 (85.0)	3 (15.0)	100	86.7	93.4
Polymyxin B + levofloxacin	19 (95.0)	1 (5.0)	95.0	85.0	90.0
Rifampicin + tigecycline	13 (65.0)	7 (35.0)	92.7	84.6	88.7
Rifampicin + meropenem	14 (70.0)	6 (30.0)	100	96.9	98.5
Rifampicin + levofloxacin	8 (40.0)	12 (60.0)	93.3	97.8	95.6
Tigecycline + meropenem	17 (85.0)	3 (15.0)	90.7	94.1	92.4
Tigecycline + levofloxacin	18 (90.0)	2 (10.0)	97.2	95.6	96.4
Meropenem + levofloxacin	14 (70.0)	6 (30.0)	97.1	84.0	90.6

^*a*^ For each antibiotic pair, the observations, sensitivity, specificity and unweighted accuracy of the bioluminescence assay were determined collectively for the 2-drug combination as well as the respective single drugs.

^*b*^ The established T_RLU_ values employed corresponded to the T_RLU_ values shown in Table 1.

## Discussion

We developed an ATP bioluminescent combination testing assay to determine effective antibiotic combinations within 24h. We found that our assay is reproducible, robust and can identify the response of CR-GNB to multiple different antibiotic combinations with a high degree of sensitivity and specificity. Potentially, this method may allow us to replace the lengthy and often laborious plating processes of viable count determination, reducing turn-around time from 3–5 days to 24h.

The use of bioluminescence-based ATP testing to detect and quantify bacteria has been described since the 1960s [29]. First described by Chappelle *et al* in 1968, bioluminescence-based ATP testing has since been well-established in the food processing industry, as well as in infection control in healthcare settings [8,29,30]. A relatively smaller number of studies have applied ATP bioluminescence in antimicrobial susceptibility testing [6,7,9]. In a study by Lafond *et al*, ATP bioluminescence was compared to standard antimicrobial susceptibility testing methods in the determination of antimicrobial susceptibilities in *Escherichia coli* and *Staphylococcus aureus* [9]. The study found that the 6h ATP bioluminescence measurements showed good concordance to conventional susceptibility testing, and was sensitive, rapid and reproducible. Similarly promising results were mirrored in other studies, which reinforced the potential for the ATP bioluminescence as a rapid method for determining the efficacy of antimicrobial agents against different bacteria [6,7].

Our study builds on prior research done to apply ATP bioluminescence to bacterial susceptibility testing. To develop the assay, we first determined the operating range of the bioluminescence assay, and demonstrated a linear relationship between the log count of viable bacteria and the log of the light output, even in CR-GNB. We observed that the lower limit of the operating range was approximately 10^3^–10^4^ CFU/ml, which precluded the correlation of ATP bioluminescence measurement to bactericidal activity of the antibiotic combinations (defined as ≥3 log_10_ CFU/ml reduction from baseline inoculum). To detect regrowth due to heteroresistance, a phenomenon previously described in XDR-GNB and has been implicated in lack of bactericidal activity at 24 h despite initial drop in bacterial inoculum, we opted to perform bioluminescence measurements after an incubation time of 24 h, which is in contrast to the incubation times of 2 to 6 h as published in previous studies [31].

We found that our ATP-bioluminescence assay distinguished between inhibitory and non-inhibitory antibiotic combinations with high accuracy compared to the conventional viable plating method. Interestingly, when individualized thresholds were determined for each species and for each antibiotic combination, differences in T_RLU_, with variations in accuracy, were observed. Most notably, the T_RLU_ of *P*. *aeruginosa* was evidently different from that of *K*. *pneumoniae* and *A*. *baumannii*, suggesting variability in ATP content among different bacterial species. As ATP is the energy currency required for bacterial growth and division, we postulate the low ATP content in *P*. *aeruginosa* to may be related to its slower growth dynamics as compared to other bacteria, which has been demonstrated in previously published studies [32]. Differences in enzymatic activity between each species may also have contributed to variability in cellular ATP content among GNB [33]. Secondly, we noted that tigecycline-containing combinations had lower unweighted accuracy compared to other combinations; of note, low accuracy (~70%) was observed for tigecycline in combination with rifampicin for *A*. *baumannii* upon internal and external validation. This may be attributable to the fact that tigecycline is intrinsically bacteriostatic against GNB; consequently, the bacterial load determined by viable plating may be an underestimation of actual bacterial cell counts in the sample [34]. This suggested that while the ATP bioluminescence method is promising, it may be less useful in determining effective combinations involving bacteriostatic agents. Variations in accuracy of the ATP-based bioluminescence assay may also be accounted by the formation of spheroplasts or filaments, which are osmotically fragile bacterial cell with high ATP content, typically induced by exposure to beta-lactam antibiotics [5,10].

To the best of our knowledge, this is the first study describing the use of ATP bioluminescence in determination of effective antibiotic combinations against CR-GNB. In a similarly designed study by Ivancic *et al*, an ATP bioluminescence assay was developed to rapidly determine antimicrobial susceptibility of uropathogens in clinical urine samples [6]. In their study, the ATP bioluminescent method demonstrated poor overall unweighted accuracy (58%) compared to conventional susceptibility testing when thresholds were not individualized for each antibiotic; when separate thresholds for each antibiotic were used, however, accuracy improved to 91% [6]. This is in contrast to the findings of our study, which demonstrated high overall unweighted accuracy even when thresholds were not individualized for each antibiotic combination or CR-GNB species. Furthermore, their study only determined optimal threshold values (internal validation); in comparison, we validated our assay through internal and external validation, further demonstrating the robustness and generalizability of our assay.

Despite our promising findings, this study has a few limitations. Firstly as mentioned earlier, our assay measured total bacterial ATP, which may have included ATP from spheroplasts or protoplasts induced by exposure to beta-lactam antibiotics. Approaches to release intracellular ATP from these filamentous cells, such as methods previously described by Hattori *et al*, may be explored in our future studies to further improve the predictive accuracy of our assay [10]. Secondly, while an incubation time of 24h was intentionally chosen to facilitate detection of any possible bacterial regrowth, we acknowledge that ATP measurement at 24h may not be optimal as the bacteria may be in stationary phase, with a resultant reduction in ATP concentrations in each bacterial cell. Furthermore, different incubation time may be required for different specimens, due to variations in doubling time. To address this shortcoming, we plan to measure bioluminescence over time in future studies, to determine the incubation time which provides the best accuracy in distinguishing inhibitor and non-inhibitory antibiotic combinations when compared to viable count.

## Conclusion

The lack of new antibiotics in the developmental pipeline has compelled physicians to adopt antibiotic combinations for the treatment of XDR-GNB. Unfortunately to date, a laboratory method that can determine combination antimicrobial susceptibility profiles in a timely manner is yet to be available routinely. We developed a rapid combination antimicrobial susceptibility testing assay using adenosine triphosphate bioluminescence, in hope of providing guidance in combination selection in a timely manner. Future studies will be carried out to refine the method, as well as to expand its utility in additional combinations against XDR-GNB.

## References

[1] Boucher HW, Talbot GH, Bradley JS, Edwards JE, Gilbert D, Rice LB, et al Bad bugs, no drugs: no ESKAPE! An update from the Infectious Diseases Society of America. Clin Infect Dis. 2009; 48: 1–12. 10.1086/595011 19035777

[2] Nordmann P, Dortet L, Poirel L Carbapenem resistance in Enterobacteriaceae: here is the storm! Trends Mol Med. 2012; 18: 263–272. 10.1016/j.molmed.2012.03.003 22480775

[3] Zavascki AP, Bulitta JB, Landersdorfer CB Combination therapy for carbapenem-resistant Gram-negative bacteria. Expert Rev Anti Infect Ther. 2013; 11: 1333–1353. 10.1586/14787210.2013.845523 24191943

[4] van Belkum A, Durand G, Peyret M, Chatellier S, Zambardi G, Schrenzel J, et al Rapid clinical bacteriology and its future impact. Ann Lab Med. 2013; 33: 14–27. 10.3343/alm.2013.33.1.14 23301218 PMC3535192

[5] Hanberger H, Svensson E, Nilsson M, Nilsson LE, Hornsten EG, Maller R Effects of imipenem on Escherichia coli studied using bioluminescence, viable counting and microscopy. J Antimicrob Chemother. 1993; 31: 245–260. 8463170 10.1093/jac/31.2.245

[6] Ivancic V, Mastali M, Percy N, Gornbein J, Babbitt JT, Li Y, et al Rapid antimicrobial susceptibility determination of uropathogens in clinical urine specimens by use of ATP bioluminescence. J Clin Microbiol. 2008; 46: 1213–1219. 10.1128/JCM.02036-07 18272708 PMC2292911

[7] Kapoor R, Yadav JS Development of a rapid ATP bioluminescence assay for biocidal susceptibility testing of rapidly growing mycobacteria. J Clin Microbiol. 2010; 48: 3725–3728. 10.1128/JCM.01482-10 20720030 PMC2953127

[8] Shama G, Malik DJ The uses and abuses of rapid bioluminescence-based ATP assays. Int J Hyg Environ Health. 2013; 216: 115–125. 10.1016/j.ijheh.2012.03.009 22541898

[9] Lafond M, Vidal N, Letourneux Y, Brunel JM A comparison of three rapid and accurate bioluminescent antibiotic susceptibility tests. J Pharmacol Toxicol Methods. 2010; 61: 16–19. 10.1016/j.vascn.2009.10.004 19861166

[10] Hattori N, Nakajima MO, O'Hara K, Sawai T Novel antibiotic susceptibility tests by the ATP-bioluminescence method using filamentous cell treatment. Antimicrob Agents Chemother. 1998; 42: 1406–1411. 9624485 10.1128/aac.42.6.1406 PMC105613

[11] Clinical and Laboratory Standards Institute. (2014) Performance Standards for Antimicrobial Susceptibility Testing. CLSI Wanyne, Pennsylvania, USA.

[12] Lim TP, Tan TY, Lee W, Sasikala S, Tan TT, Hsu LY, et al In-vitro activity of polymyxin B, rifampicin, tigecycline alone and in combination against carbapenem-resistant Acinetobacter baumannii in Singapore. PLoS One. 2011; 6: e18485 10.1371/journal.pone.0018485 21533030 PMC3080872

[13] Teo J, Cai Y, Tang S, Lee W, Tan TY, Tan TT, et al Risk factors, molecular epidemiology and outcomes of ertapenem-resistant, carbapenem-susceptible Enterobacteriaceae: a case-case-control study. PLoS One. 2012; 7: e34254 10.1371/journal.pone.0034254 22461908 PMC3312905

[14] Balm MN, Ngan G, Jureen R, Lin RT, Teo J Molecular characterization of newly emerged blaKPC-2-producing Klebsiella pneumoniae in Singapore. J Clin Microbiol. 2012; 50: 475–476. 10.1128/JCM.05914-11 22116160 PMC3264177

[15] Roy S, Datta S, Viswanathan R, Singh AK, Basu S Tigecycline susceptibility in Klebsiella pneumoniae and Escherichia coli causing neonatal septicaemia (2007–10) and role of an efflux pump in tigecycline non-susceptibility. J Antimicrob Chemother. 2013; 68: 1036–1042. 10.1093/jac/dks535 23335112

[16] Kitchel B, Rasheed JK, Endimiani A, Hujer AM, Anderson KF, Bonomo RA, et al Genetic factors associated with elevated carbapenem resistance in KPC-producing Klebsiella pneumoniae. Antimicrob Agents Chemother. 2010; 54: 4201–4207. 10.1128/AAC.00008-10 20660684 PMC2944623

[17] Tod M, Lortholary O, Seytre D, Semaoun R, Uzzan B, Guillevin L, et al Population pharmacokinetic study of amikacin administered once or twice daily to febrile, severely neutropenic adults. Antimicrob Agents Chemother. 1998; 42: 849–856. 9559795 10.1128/aac.42.4.849 PMC105554

[18] Rebuck JA, Fish DN, Abraham E Pharmacokinetics of intravenous and oral levofloxacin in critically ill adults in a medical intensive care unit. Pharmacotherapy. 2002; 22: 1216–1225. 12389872 10.1592/phco.22.15.1216.33484

[19] Gumbo T, Louie A, Deziel MR, Liu W, Parsons LM, Salfinger M, et al Concentration-dependent Mycobacterium tuberculosis killing and prevention of resistance by rifampin. Antimicrob Agents Chemother. 2007; 51: 3781–3788. 17724157 10.1128/AAC.01533-06 PMC2151424

[20] Zavascki AP, Goldani LZ, Cao G, Superti SV, Lutz L, Barth AL, et al Pharmacokinetics of intravenous polymyxin B in critically ill patients. Clin Infect Dis. 2008; 47: 1298–1304. 10.1086/592577 18840079

[21] Rodvold KA, Gotfried MH, Cwik M, Korth-Bradley JM, Dukart G, Ellis-Grosse EJ Serum, tissue and body fluid concentrations of tigecycline after a single 100 mg dose. J Antimicrob Chemother. 2006; 58: 1221–1229. 17012300 10.1093/jac/dkl403

[22] Jaruratanasirikul S, Sriwiriyajan S, Punyo J Comparison of the pharmacodynamics of meropenem in patients with ventilator-associated pneumonia following administration by 3-hour infusion or bolus injection. Antimicrob Agents Chemother. 2005; 49: 1337–1339. 15793108 10.1128/AAC.49.4.1337-1339.2005 PMC1068632

[23] Wadhwani T, Desai K, Patel D, Lawani D, Bahaley P, Joshi P, et al Effect of various solvents on bacterial growth in context of determining MIC of various antimicrobials. The Internet Journal of Microbiology. 2008; 7.

[24] Lim TP, Lee W, Tan TY, Sasikala S, Teo J, Hsu LY, et al Effective antibiotics in combination against extreme drug-resistant Pseudomonas aeruginosa with decreased susceptibility to polymyxin B. PLoS One. 2011; 6: e28177 10.1371/journal.pone.0028177 22162759 PMC3230594

[25] Pournaras S, Vrioni G, Neou E, Dendrinos J, Dimitroulia E, Poulou A, et al Activity of tigecycline alone and in combination with colistin and meropenem against Klebsiella pneumoniae carbapenemase (KPC)-producing Enterobacteriaceae strains by time-kill assay. Int J Antimicrob Agents. 2011; 37: 244–247. 10.1016/j.ijantimicag.2010.10.031 21236643

[26] Vouillamoz J, Moreillon P, Giddey M, Entenza JM In vitro activities of tigecycline combined with other antimicrobials against multiresistant gram-positive and gram-negative pathogens. J Antimicrob Chemother. 2008; 61: 371–374. 18033780 10.1093/jac/dkm459

[27] Aaron SD, Ferris W, Henry DA, Speert DP, Macdonald NE Multiple combination bactericidal antibiotic testing for patients with cystic fibrosis infected with Burkholderia cepacia. Am J Respir Crit Care Med. 2000; 161: 1206–1212. 10764313 10.1164/ajrccm.161.4.9907147

[28] Magiorakos AP, Srinivasan A, Carey RB, Carmeli Y, Falagas ME, Giske CG, et al Multidrug-resistant, extensively drug-resistant and pandrug-resistant bacteria: an international expert proposal for interim standard definitions for acquired resistance. Clin Microbiol Infect. 2011. 10.1111/j.1469-0691.2011.03570.x 21793988

[29] Chappelle EW, Levin GV Use of the firefly bioluminescent reaction for rapid detection and counting of bacteria. Biochem Med. 1968; 2: 41–52.

[30] Amodio E, Dino C Use of ATP bioluminescence for assessing the cleanliness of hospital surfaces: a review of the published literature (1990–2012). J Infect Public Health. 2014; 7: 92–98. 10.1016/j.jiph.2013.09.005 24231159

[31] Li J, Rayner CR, Nation RL, Owen RJ, Spelman D, Tan KE, et al Heteroresistance to colistin in multidrug-resistant Acinetobacter baumannii. Antimicrob Agents Chemother. 2006; 50: 2946–2950. 16940086 10.1128/AAC.00103-06 PMC1563544

[32] Lee DH, Koh EH, Choi SR, Kim S Growth dynamics of Staphylococcus aureus, Escherichia coli, and Pseudomonas aeruginosa as a function of time to detection in BacT/alert 3D blood culture bottles with various preincubation conditions. Ann Lab Med. 2013; 33: 406–409. 10.3343/alm.2013.33.6.406 24205488 PMC3819438

[33] Funke G, Monnet D, deBernardis C, von Graevenitz A, Freney J Evaluation of the VITEK 2 system for rapid identification of medically relevant gram-negative rods. J Clin Microbiol. 1998; 36: 1948–1952. 9650942 10.1128/jcm.36.7.1948-1952.1998 PMC104958

[34] Pankey GA Tigecycline. J Antimicrob Chemother. 2005; 56: 470–480. 16040625 10.1093/jac/dki248

